# OUTPATIENT ANALYTIC ASSESSMENT OF ANOREXIA NERVOSA — THE IMPORTANCE OF VENOUS BLOOD GASES

**DOI:** 10.1590/1984-0462/2020/38/2018358

**Published:** 2020-01-13

**Authors:** Sofia Alexandra Pereira Pires, Joana Costa Soares, Alexandra Maria Branco da Luz, Pascoal Moleiro

**Affiliations:** a Centro Hospitalar e Universitário de Coimbra, Coimbra, Portugal.; b Centro Hospitalar de Leiria, Leiria, Portugal.

**Keywords:** Anorexia nervosa, Adolescent, Malnutrition, Blood gas analysis, Anorexia nervosa, Adolescente, Desnutrição, Gasometria

## Abstract

**Objective:**

To evaluate serum biochemical parameters’ evolution, especially venous blood gas (VBG), in anorexia nervosa (AN), correlating with clinical parameters.

**Methods:**

Retrospective study including out-patient AN adolescents, between January 2014 and May 2017. Three evaluations were compared: t1) first consultation; t2) consultation with the lowest body mass index (BMI) z-score and t3) with the highest BMI z-score.

**Results:**

A total of 24 adolescents (87.5% females) were included, mean age of presentation of 14.9±1.7 years, onset of symptoms 6.4±3.2 months before the first visit. In t1, BMI z-score of -1.91±1.11 kg/m^2^ and ideal weight % of 84.3±9.2. Amenorrhea was present in 88%. In t2 the analytical alterations were: altered VBG in 100%, altered ferritin (72% elevated), altered thyroid function (53% with thyroxine decrease), dyslipidemia (31% elevation of high density lipoprotein, 25% hypercholesterolemia), elevation of urea (25%), elevation of alanine aminotransferase (14%), hypoglycemia (14%), anemia (9%). Respiratory acidosis was present in 91% in t1, 100% in t2 and 94% in t3. There was a significant decrease between t2 and t3 in mean pCO^2^ (57.2 *versus* 53.6 mmHg; p=0.009) and mean HCO_3_ (30.0 *versus* 28.8 mEq/L; p=0.023).

**Conclusions:**

Respiratory acidosis and increased ferritin were common in this group. Respiratory acidosis was the most frequent abnormality with significant pCO2 and HCO_3_ variation in the recovery phase. VBG should be considered in AN evaluation, once it seems to be important in assessing the severity of the disease and its subsequent follow-up.

## RESUMO

**Objetivo:**

Avaliar a evolução laboratorial, particularmente da gasometria venosa, na anorexia nervosa (AN), correlacionando os achados com parâmetros clínicos.

**Métodos:**

Estudo retrospetivo com adolescentes com AN seguidos em ambulatório, entre janeiro de 2014 e maio de 2017. Foram comparadas três avaliações: (t1) primeira consulta; (t2) consulta com escore Z de índice de massa corpórea (IMC) mais baixo; e (t3) consulta com escore Z de IMC mais elevado.

**Resultados:**

Incluídos 24 adolescentes, 87,5% do sexo feminino, idade média de apresentação de 14,9±1,7 anos, início dos sintomas 6,4±3,2 meses antes da primeira consulta. Em t1, escore Z de IMC de -1,91±1,11 kg/m^2^ e % de peso ideal de 84,3±9,2. Tinham amenorreia 88%. Em t2 as alterações laboratoriais encontradas foram: gasometria venosa alterada em 100%, ferritina alterada (72% elevada), função tiroideia alterada (53% com diminuição da tiroxina), dislipidemia (31% com elevação de lipoproteína de alta densidade, 25% com hipercolesterolemia), elevação da ureia (25%), elevação da alanina aminotransferase (14%), hipoglicemia (14%) e anemia (9%). A acidose respiratória esteve presente em 91% em t1, 100% em t2 e 94% em t3. Verificou-se diminuição significativa entre t2 e t3 da pressão parcial de CO_2_ (pCO_2_) média (57,2 *versus* 53,6 mmHg; p=0,009) e HCO_3_ médio (30,0 *versus* 28,8 mEq/L; p=0,023).

**Conclusões:**

A acidose respiratória e o aumento da ferritina foram comuns nesse grupo. Acidose respiratória foi a alteração mais frequente, com variação significativa de pCO2 e HCO_3_ na fase de recuperação. A gasometria venosa deve ser considerada na avaliação laboratorial na AN, pois parece ser importante na avaliação da gravidade e monitorização da doença.

## INTRODUCTION

Anorexia nervosa (AN) is a multifactorial eating disorder with 0.5 to 2% prevalence in the general population. It is more frequent among females and its incidence peak of disease onset is between 13 and 18 years.^1-5^ AN-associated mortality is 5 to 6%, the highest rate for women by a psychiatric illness.^3^

Since 2013, its diagnosis has been based on the criteria of the Diagnostic and Statistical Manual of Mental Disorder V (DSM-5),^6^ divided into restrictive and compulsive/purgative subtypes. This classification allowed to decrease the prevalence of diagnosis of eating disorder without further specification, to the detriment of the increase in AN cases, allowing for greater therapeutic and prognostic accuracy.^7^

The combination of malnutrition implicated in AN and potentially associated behaviors, such as vomiting and use of laxatives, may impact various organs and systems, and be associated with various laboratory abnormalities.^8-10^ The most commonly described laboratory abnormalities are: electrolyte disorders such as hypophosphatemia and hypokalemia due to vomiting and the use of laxative/diuretic substances.^2,8^ Endocrine dysfunction is relatively frequent and includes hypothyroidism, hypercortisolism, and hypothalamus-pituitary axis disorders manifested by hypogonadotropic hypogonadism. and anovulation.^9^ The most common alteration is the euthyroid syndrome (low levels of thyroxine with normal thyrotropin), reversible with reestablishment of feeding.^1^ Patients may also have impaired kidney function associated with acute renal failure due to vomiting or severe fluid restriction.^2^ Other changes are hematological disorders such as anemia and leukopenia, with rare cases of thrombocytopenia. It is important to assess iron and vitamin B12 levels in anemic patients because supplementation is recommended in cases of deficiency.^2^ Elevation of transaminases is common, usually asymptomatic and self-limiting, but in rare cases may be associated with liver injury.^8^ Mild hypoglycaemia is a common finding in AN and is usually well tolerated.^8^ Serum albumin is normal in most patients, even in severe cases.^8^ Several studies have shown elevated levels of total cholesterol (TC), high density lipoprotein (LDL) and low density lipoprotein (HDL). The cause is not fully understood, but there are studies linking this finding to reduced catabolism and decreased thyroxine (T3).^8^ Changes in acid-base balance, such as metabolic alkalosis in patients with purgative or behavioral disorders, are also described in patients who make use of diuretics.^1,8,11^ Studies in hospitalized patients have shown that respiratory acidosis is also a common finding in AN.^11,12^ However, blood gas analysis is not part of the routine laboratory evaluation of most centers, particularly in outpatient follow-up.

Several laboratory parameters related to nutritional status are routinely evaluated after the diagnosis of AN.^1,13^ However, even in severe cases, laboratory evaluation may not change.^14^ The explanation for this fact seems to lie in the compensation. of the effects of malnutrition, which preserves most physiological functions through adaptive mechanisms,^14,15^ although clinical parameters of malnutrition are present.

Most longitudinal studies on laboratory abnormalities in AN patients are performed with hospitalized patients,^11,14-16^ in whom a more pronounced metabolic imbalance can be predicted. There are few studies analyzing the evolution of laboratory parameters in patients with AN followed up in outpatient clinic^10,17^ that allow to infer their role in risk stratification and clinical monitoring of these patients.

Establishing a laboratory parameter that correlates with clinical outcome/severity is important for therapeutic monitoring and also for patient awareness of the need for treatment. Thus, the objective of this study was to evaluate the evolution of laboratory parameters, particularly venous blood gas analysis in AN patients followed up at an outpatient clinic, and relate them with clinical parameters.

## METHOD

An analytical cross-sectional study was conducted with retrospective data collection from a convenience sample. Data were analyzed from clinical records of adolescents with AN diagnosed based on DSM-5 criteria and followed up at the adolescent medicine consultation of a level II hospital, between January 2014 and May 2017.

Three points were considered for the evaluation of anthropometric and laboratory parameters: (t1) first consultation; (t2) consultation with the lowest body mass index (BMI) Z-score; and (t3) consultation with the highest BMI Z-score. In some cases, the consultation with the lowest BMI Z-score (t2) corresponded to the first consultation (t1).

At each of these assessment points the following anthropometric data were analyzed: weight, height, BMI, BMI percentile, BMI Z-score and % of ideal weight. The BMI percentile and Z-score were calculated using the BMI calculator for children and adolescents, available at https://zscore.research.chop.edu/index.php. To determine the ideal weight of adolescents, the weight corresponding to BMI at the 50th percentile (P50) for age and sex was calculated using the formula: target weight (kg) = BMI at P50 (kg/m^2^) × height (meters).^2^ The % of ideal weight was calculated using the formula: actual weight × 100/target weight.

At the three assessment points, blood pressure (BP) and heart rate (HR) were also evaluated. Bradycardia was considered when HR<60 bpm and hypotension when systolic BP<90 mmHg.

The laboratory parameters analyzed were: hemoglobin (g/dL), sodium (mmol/L), potassium (mmol/L), magnesium (mmol/L), phosphate (mmol/L), calcium, total cholesterol, triglycerides, LDL, HDL, creatinine kinase (CK) (U/L), albumin, creatinine (mg/dL), urea (mmol/L), thyrotropin (TSH), free T4, total protein (g/dL), glucose (mg/ dL), aspartate aminotransferase (AST) (U/L), alanine aminotransferase (ALT) (U/L), lactic dehydrogenase (LDH) (U/L), ferritin (ng/mL) and pH, CO_2_ partial pressure (pCO_2_) and HCO_3_ (venous blood gas analysis). Like other authors,^11^ in this study we used arterial blood gas reference values to analyze venous blood gas results. Some authors^18,19^ have shown a high degree of agreement between arterial and venous blood gas values.

Considering the ease of obtaining a venous blood sample, there is no theoretical or practical reason to justify the need to use arterial blood gas in AN patients. As reference values for the remaining analyses, internationally accepted values were considered at the pediatric age,^20^ as well as the reference values for pediatric age for dyslipidemia, from the Portugal General Directorate for Health.^21^ Data were also analyzed as age at first visit, gender, duration of disease, presence of amenorrhea and severity of AN (mild: BMI ≥17 kg/m^2^; moderate: BMI 16 to 16.9 kg/m^2^; severe: BMI 15 to 15.9 kg/m^2^; extreme: BMI < 15 kg/m^2^).

For comparative analysis, the Student’s t-test for paired samples was used to compare means and the McNemar’s test for categorical variables. To investigate the correlation between quantitative variables, we used the Pearson’s (r) or Spearman’s (rho) coefficient, according to normality. The absolute value of correlation indicates the intensity of the association, considering r/rho≤0.19: very low correlation; 0.2≤r/rho≤0.39: low correlation; 0.4≤r/rho≤0.69: moderate correlation; 0.7≤r/rho≤0.89: strong correlation; 0.9≤r/rho≤1: very strong correlation.^22^ Statistical treatment was performed using the Statistical Package for Social Sciences (SPSS) 22^®^ (IBM Corp. Armonk, NY, USA). Statistical significance was set for p<0.05.

The survey and consultation of clinical records were made respecting the confidentiality of adolescents’ data; and data collection complied with the principles of the Declaration of Helsinki. The study was authorized by the service director, as well as by the Hospital Research Ethics Committee.

## RESULTS

A sample of 24 adolescents was obtained, 21 participants being females (87.5%). Three patients were seen at only one consultation during the study and, therefore, have only the laboratory evaluation for t1.

At their first appointment, the patients had a mean age of 14.9±1.7 years and onset of symptoms 6.4±3.2 months earlier.

The ideal weight percentage was 84.3±9.2%; the BMI percentile was 13.2±16.4; and the BMI Z-score was -1.91±1.11. Regarding AN severity, 66.7% of the sample had mild to moderate AN, with no statistically significant differences regarding gender (p=0.526). Note that 4 patients (16.7%) had a BMI <15 kg/m^2^, which corresponds to a degree of extreme severity.

Of female adolescents with menarche (n=17), 88% had secondary amenorrhea.

As for the vital parameters evaluated, 62.5% presented bradycardia and 12.5% had hypotension. Patients with severe and extreme AN presented bradycardia and hypotension more often than patients with mild to moderate AN (87.5 versus 50%; p=0.074; and 25 versus 6.3%; p=0.19, respectively). Table 1 shows the characteristics of the sample upon first consultation.

**Table 1:** Sample characterization at the first appointment (n = 24).

Parameters	First appointment
Age (years)	14.9±1.7 (variation 11–18)
Female/male (n)	21/3
Time since symptoms onset (months)	6.4±3.2 (variation 2–12)
Ideal weight %	84.3±9.2
BMI Z-score	-1.91±1.11
Severity^a^	
Mild	9
Moderate	7
Severe	4
Extreme	4
Amenorrhea (with menarche n=17)	15 (88%)
HR (bpm)	
Mean±SD	58.3±13.7
% bradycardia	62.5
Systolic BP (mmHg)	
Mean±SD	99.3±9.0
% hypotension	12.5

BMI: body mass index; HR: heart rate; SD: standard deviation; BP: blood pressure; ^a^American Psychiatric Association.^6^

Between t2 and t3, the increase in BMI Z-score was accompanied by a significant increase in HR (60.1±13.4 versus 70.8±14.6; p=0.022) and BP (97.3±10.1 versus 102.2±11.2; p=0.031), as shown in Table 2.

**Table 2:** Characterization of vital parameters and association with body mass index.

Parameters	t1	t2	t3	p-value^a^
BMI score (Mean±SD)	-1.65±1.13	-2.41±1.31	-0.85±0.90	<0.001
HR (bpm)				
Mean±SD	58.3±13.7	60.1±13.4	70.8±14.6	0.022
% bradycardia	62.5	60	23.8	
Systolic BP (mmHg)				
Mean±SD	99.3±9.0	97.3±10.1	102.2±11.2	0.031
% hypotension	12.5	20	10	

BMI: body mass index; SD: standard deviation; HR: heart rate; BP: blood pressure; ^a^Student’s t-test for paired samples (between means of t2 and t3).

The exams requested in each laboratory evaluation (t1, t2 and t3) varied, with blood count and glucose requested in all evaluations. In addition to these, the most frequent tests were: urea, creatinine, TSH, free T4, complete ionogram, lipid profile, transaminases and venous blood gases, requested in more than 75% of cases. Their analysis is presented in Table 3.

**Table 3:** Laboratory parameters of patients with anorexia nervosa at the three moments of analysis.

	Reference values	t1 (n=24) (mean±SD) (normal value in %)	t2 (n=21) (mean±SD) (normal value in %)	t3 (n=21) (mean±SD) (normal value in %)	p-value^a^
Hb (g/dL)	M (12.5–16.1) F (12–15)	13.6±1.5 88	13.4±1.6 86	13.1±1.2 81	0.395
Glucose (mg)	60–100	75.5±12.1 83	76.2±15.7 81	79.2±10.9 86	0.438
Total proteins (g/dL)	66-82	74.3±4.5 95	72.9±5.3 94	72.8±3.6 93	0.735
AST (U/L)	10–15Y(10–40) 16–19Y(15–45)	25.2±7.4 96	24.1±5.6 100	25.9±6.7 100	0.137
ALT (U/L)	5–45	26.3±11.7 92	27.5±12.9 86	32.7±21.7 85	0.366
LDH (U/L)	120–330	208.1±47.7 100	183.7±27.0 100	185.5±20.8 100	0.973
CK (U/L)	5–130	97.8±45.8 75	68.7±20.9 100%	72.4±26.8 100	0.715
Urea (mmol/L)	2.5–6.4	6.2±3.9 75	5.3±1.4 75	6.4±4.2 76	0.224
Creatinine (mg/dL)	10–14Y (0.31–0.88) 15–19Y (0.5–1.06)	0.8±0.2 96	0.7±0.1 100	0.7±0.1 95	0.051
HDL (mg/dL)	>35	58.4±11.3 100	57.0±9.23 100	61.3±14.6 100	0.332
LDL (mg/dL)	<130	98.6±39.4 80	99.8±41.7 69	84.7±28.2 94	0.025
TC (mg/dL)	<200	165.5±53.2 70	165.0±45.4 75	151.2±47.0 89	0.026
TG (mg/dL)	<150	72.3±22.9 100	68.6±24.6 100	71.9±0.4 94%	0.999
TSH (uUI/mL)	0.5–4.5	1.8±0.8 95	1.55±0.8 94	1.7±0.7 100	0.173
T4L (pmol/L)	9–25.7	9.9±1.3 84	9.3±1.6 47	8.9±2.3 53	0.748
K (mmol/L)	3.3–4.6	4.3±0.3 96	4.0±0.3 100	4.1±0.3 90	0.257
Na (mmol/L)	134–145	139.3±1.6 100	140.0±1.6 100	139.7±2.7 100	0.419
Ca (mmol/L)	2.2–2.7	2.4±0.4 86	2.3±0.5 79	2.2±0.4 89%	0.924
Mg (mmol/L)	0.6–0.95	0.9±0.1 77%	0.9±0.1 88	0.8±0.1 94	0.401
Phosphate (mmol/L)	12–15Y: 0.95–1.75; 16–19Y: 0.9–1.5	1.3±0.2 100	1.3±0.2 100	1.3±0.2 100	0.975
Ferritin (ng/mL)	M: 10–300 F: 10–70	160.8±159.9 (F: 163.2±169.4)^b^ 10	191.7±182.7 (F: 191.6±182.7)^b^ 14	70.0±39.6 (F: 74.2±40.9)^b^ 37	0.196 (F 0.196)
Venous pH	7.35–7.45	7.34±0.03^b^ 59	7.33±0.02^b^ 30	7.34±0.02^b^ 47	0.213
pCO_2_ (mmHg)	<45	53.8±6.5^b^ 5	56.6±5.1^b^ 0	53.5±5.2^b^ 6	0.009
HCO_3_ (mmHg)	22–29 (venous) 21–28 (arterial)	28.9±2.0 36	29.9±1.6 25	28.7±2.5 47	0.023

SD: standard deviation; Hb: hemoglobin; AST: aspartate aminotransferase; Y: Years ago; ALT: alanine aminotransferase; LDH: lactate dehydrogenase; CK: creatinine kinase; HDL: high density lipoprotein; LDL: low density lipoprotein; TC: total cholesterol; TG: triglycerides; TSH: thyrotropin; T4L: free thyroxine; K: potassium; Na: sodium; Ca: calcium; Mg: magnesium; pCO_2_: CO_2_ partial pressure; HCO_3_: bicarbonate; F: female; M: male; ^a^Student’s t-test for paired samples (between t2 and t3 average); ^b^means differing from reference values.

Considering the laboratory evaluation performed with the lowest BMI (t2), most laboratory parameters were normal. Changes found were: venous blood gas analysis (100% pCO_2_ elevation, 75% HCO_3_ elevation, 70% acidosis), ferritin (72% elevated, 14% decreased), thyroid function (53% decreased thyroxine, 6% decreased thyrotropin), dyslipidemia (31% with elevated LDL, 25% with hypercholesterolemia), elevated urea (25%), elevated ALT (14%), hypoglycemia (14%) and anemia (9%).

Considering the mean laboratory parameters, venous blood gas and ferritin (in females) were the only altered parameters (Table 3). Ferritin was high in t1 and t2 (163.2 and 191.6 ng/mL, respectively), considering the reference values for females. Its value decreased between t2 and t3, without statistical significance (p=0.196). No significant correlation was found between ferritin values and BMI Z-score. Although the lipid profile values were within the limits established as reference, there was a significant decrease, between t2 and t3, of the mean total cholesterol (167.4 versus 148.5 mg/dL; p=0.026) and LDL (98.4 versus 83.8 mg/dL; p=0.025).

There is a positive correlation between BMI Z-score variation and mean glucose variation between t2 and t3 (rho=0.464; p=0.034).

Venous blood gas results are shown in Table 4. Respiratory acidosis (compensated or not) was present in 20 patients at t1 (91%), 20 patients at t2 (100%) and 16 patients at t3 (94%). The blood gas parameters at first visit did not correlate with the duration of disease or with the Z score at t1. There was a decrease in the number of unbalanced acidoses from t2 to t3 (without statistical significance; p=0.453). There was also a significant decrease between t2 and t3 of mean pCO_2_ (57.2 versus 53.6mmHg; p=0.009) and mean HCO_3_ (30.0 versus 28.8 mEq/L; p=0.023).

**Table 4:** Venous blood gas analysis.

Venous blood gas analysis	t1 (n=22)	t2 (n=20)	t3 (n=17)	p-value
pH	7.34±0.03^a^	7.33±0.02^a^	7.34±0.02^a^	0.213^b^
pCO_2_ (mmHg)	53.8±6.5^a^	56.6±5.1^a^	53.5±5.2^a^	0.009^b^
HCO_3_ (mEq/L)	28.9±2.0	29.9±1.6	28.7±39.7	0.230^b^
Respiratory acidosis	20 (91%)	20 (100%)	16 (95%)	NA
Compensated respiratory acidosis (pH 7.35–7.45 e pCO_2_>45)	11 (50%)	7 (35%)	7 (41%)	NA
Non-compensated respiratory acidosis (pH <7.35 e pCO_2_>45)	9 (41%)	13 (65%)	9 (53%)	0.453^c^

pCO_2_: CO_2_ partial pressure; HCO_3_: bicarbonate; NA: not applicable; ^a^altered means related to reference values; ^b^Student’s t-test for paired samples (between t2 and t3 mean); ^c^McNemar test (between t2 and t3).

## DISCUSSION

In this study, most of the laboratory parameters usually requested for patients with anorexia nervosa presented means values within the reference range, except for venous blood gas and ferritin parameters. At the greatest clinical severity (t2 – lowest BMI and most often presence of bradycardia and hypotension), the most frequent laboratory abnormalities were found in venous blood gas analysis. In order of frequency, ferritin alterations (72% with elevation), altered thyroid function (53% with decreased thyroxine), dyslipidemia (31% with elevated LDL, 25% with hypercholesterolemia), elevated urea (25%), increased ALT (14%), hypoglycemia (14%) and anemia (9%) were also found.

There is great variability in the results of studies evaluating laboratory changes in AN, due to the heterogeneity of the methodology used (laboratory parameters analyzed, inpatients/ outpatients, comparison with reference values/control groups). Nova et al.^14,15^ analyzed hospitalized (and probably more decompensated) patients compared to a control group and described frequent laboratory abnormalities, especially in blood count, blood glucose, total proteins, transaminases, LDH, CK and ferritin. In these patients recruited during hospitalization, the presence of dyslipidemia was less frequent than in our study (18% hypercholesterolemia and 16% elevation of LDL).^23^ Also compared to the findings of this study, in other outpatient studies^10,17^ anemia was more frequent (38.6%), as were hydroelectrolytic changes (hyponatremia in 19.7% and hypokalemia in 19.7%), but with similar ALT elevation values (12.2%).

These results show that laboratory alterations may be uncommon, even in severe patients. Laboratory “normality” in this disease, which clinically presents with physical signs of malnutrition in various systems, may, on the one hand, cause the less trained clinician to downplay the severity, and, on the other, endorse the patient’s behavior, who may understand that their values are within laboratory references.

As mentioned, the most frequent laboratory alterations in this sample were venous blood gas analysis and ferritin. Data in the literature regarding blood gas changes in patients with anorexia are controversial. Some studies reporte normal values,^24,25^ while others report that changes in (venous) blood gases are frequent.^11,12^ Different results may be related to different methodologies, namely related to age, disease phase (decompensated versus “stable”), and use of arterial versus venous blood gas analysis. Kerem et al.^11^ reported that mild respiratory acidosis is common in venous blood gas analysis in adolescents with recent diagnosis of AN and hospitalized for clinical stabilization (78% at admission and 35% at hospital discharge). These data show that changes in venous blood gases are also frequent in adolescents in outpatient follow-up and at different stages of the disease.

Although there was no direct correlation between BMI Z-score and venous blood gas parameters, at the consultation with the worst BMI (t2), all patients had respiratory acidosis, which is in agreement with data reported by Kerem et al.^11^ There was a decrease in the number of patients with unbalanced acidosis from t2 to t3, associated with a significant decrease of mean pCO_2_ (57.2 versus 53.6 mmHg; p=0.009) and mean HCO_3_ (30.0 versus 28.8 mEq/L; p=0.023) between t2 and t3.

This data seems to place blood gas analysis as a useful tool for disease monitoring, since BMI recovery was associated with a tendency to normalize venous blood gas values. Several physiological mechanisms may explain the presence of respiratory acidosis in AN patients: changes in muscle strength of the muscles involved in breathing (particularly the diaphragm),^25,26^ abnormalities in respiratory control (increased vagal tone)^24,26^ and possible changes in pulmonary function (emphysema-like changes)^.27^

In this sample, ferritin was altered: elevated in t1 and t2 and with tendency to normalization in t3. Compared to changes in venous blood gas analysis, ferritin elevation was less frequent and ferritin decrease was not statistically significant (p=0.196). Although these results are not consensual in the literature,^17^ several authors have reported increased ferritin values in AN patients, with a tendency to normalization associated with weight recovery.^15,16,28-30^ Ferritin is frequently used to evaluate Iron stores, but may be elevated in situations such as liver damage, neoplasia, infection, and inflammation. Thus, it is difficult to interpret its elevation in AN patients, and, currently, its pathophysiological mechanism is not clear.

In a study by Papillard-Marechal et al.,^28^ ferritin and hepcidin concentration was high in AN patients, with no evidence of iron overload or hemolysis, and with inflammatory parameters and normal liver tests. The authors suggest that acute malnutrition may be a source of stress at the hepatocyte level, leading to an increase in hepcidin and, consequently, ferritin. Nova et al.^15^ argue that ferritin increases in response to a process of adaptation to food restriction, with normalization once weight is recovered.

Our study had some limitations because it was a retrospective analysis, with a small convenience sample and few male adolescents. Considering the study period, in three cases the clinical and laboratory evaluation was made only at the first consultation, since the subsequent consultations were later than the time period considered. These were the cases of two female adolescents and one male, which does not change the overall representativeness of the sample in terms of gender, although in the context of a small sample, it accounts for 12.5% of the total.

In addition, the laboratory evaluation was not completely homogeneous at the different moments and there was no control group. Other relevant clinical information, such as laxative intake, quantification of vomiting, physical exercise, and medication or drug/alcohol/tobacco intake were not considered. It would also be important to assess respiratory rate, as it could help to understand the mechanism associated with respiratory acidosis in these patients. A prospective study with greater sampling power could surpass such limitations. It would also be interesting to include other eating disorders.

In conclusion, this study showed that changes in laboratory tests commonly requested are uncommon in adolescents with AN followed up at the outpatient clinics. On the other hand, venous blood gas analysis (in almost all patients) and ferritin (in a smaller percentage and with probable interpretation limitations) were frequently altered. Thus, it is considered that both tests should be included in the laboratory evaluation of patients with AN. Specifically, the presence of respiratory acidosis in venous blood gas analysis in these patients was associated with greater clinical severity and may be an early marker of decompensation. In the future and by further studies, venous blood gas analysis may be clinically useful in the risk stratification of outpatients with AN followed up for monitoring/recovery.
